# Cognitive trajectories from infancy to early adulthood following birth before 26 weeks of gestation: a prospective, population-based cohort study

**DOI:** 10.1136/archdischild-2017-313414

**Published:** 2017-11-16

**Authors:** Louise Linsell, Samantha Johnson, Dieter Wolke, Helen O’Reilly, Joan K Morris, Jennifer J Kurinczuk, Neil Marlow

**Affiliations:** 1 National Perinatal Epidemiology Unit (NPEU), Nuffield Department of Population Health, University of Oxford, Oxford, UK; 2 Department of Health Sciences, Centre for Medicine, University of Leicester, Leicester, UK; 3 Department of Psychology, Division of Mental Health and Wellbeing, Warwick Medical School, University of Warwick, Coventry, UK; 4 Institute of Women’s Health, University College London, London, UK; 5 Centre for Environmental and Preventive Medicine, Barts and The London School of Medicine and Dentistry, Queen Mary University of London, London, UK

**Keywords:** epidemiology, neurodevelopment, neonatology

## Abstract

**Objective:**

To determine the trajectory of cognitive test scores from infancy to adulthood in individuals born extremely preterm compared with term-born individuals.

**Design:**

A prospective, population-based cohort study.

**Setting:**

276 maternity units in the UK and Ireland.

**Patients:**

315 surviving infants born less than 26 completed weeks of gestation recruited at birth in 1995 and 160 term-born classroom controls recruited at age 6.

**Main outcome measures:**

Bayley Scales of Infant Development-Second Edition (age 2.5); Kaufman Assessment Battery for Children (ages 6/11); Wechsler Abbreviated Scale of Intelligence-Second Edition (age 19).

**Results:**

The mean cognitive scores of extremely preterm individuals over the period were on average 25.2 points below their term-born peers (95% CI −27.8 to −22.6) and remained significantly lower at every assessment. Cognitive trajectories in term-born boys and girls did not differ significantly, but the scores of extremely preterm boys were on average 8.8 points below those of extremely preterm girls (95% CI −13.6 to −4.0). Higher maternal education elevated scores in both groups by 3.2 points (95% CI 0.8 to 5.7). Within the extremely preterm group, moderate/severe neonatal brain injury (mean difference: −10.9, 95% CI −15.5 to −6.3) and gestational age less than 25 weeks (mean difference: −4.4, 95% CI −8.4 to −0.4) also had an adverse impact on cognitive function.

**Conclusions:**

There is no evidence that impaired cognitive function in extremely preterm individuals materially recovers or deteriorates from infancy through to 19 years. Cognitive test scores in infancy and early childhood reflect early adult outcomes.

**What is already known?**

- The most common neurological impairment in children born extremely preterm is in cognitive function.
- Cognitive development has been well described in infancy and early childhood, but the trajectory into early adulthood is unknown.

**What this study adds?**

- Impaired cognitive function evident in infancy persists into early adulthood among individuals born extremely preterm, with no evidence of substantial recovery or deterioration.
- Cognitive test scores in infancy and early childhood reflect early adult outcomes.
- Male sex and moderate/severe neonatal brain injury has an adverse effect on cognitive trajectories in individuals born extremely preterm.

Cognitive impairment is the most common neurological outcome in infants born extremely preterm (EP), and poor cognitive test scores at school age are strongly related to low gestational age (GA) at birth.^1–3^ Low IQ scores in childhood are associated with reduced survival and poorer health later in life.^4 5^ Studies show that cognitive ability remains relatively stable from middle childhood onward in the general population.^6 7^ Recent evidence from very preterm and very low birthweight (VP/VLBW) cohorts suggests that deficits in cognitive function and academic attainment persist into early adulthood,^8–10^ and that developmental scores from as early as 2 years are predictive of outcomes into adulthood.^11^ Little is known about the maturation of cognition over childhood and into adulthood for EP survivors. Failure to catch up by early adulthood raises concerns about the future trajectory of cognitive function in later adult life.

Studies investigating cognitive development in VP/VLBW survivors have yielded mixed findings. Some studies report deterioration over time, while others suggest that cognitive function remains stable, or even improves into adolescence relative to term-born controls.^12–15^ However, much of this evidence has come from cross-sectional analyses of longitudinal data, which may explain the inconsistent findings. There may be considerable variation in individual trajectories that is not detectable using such analytical methods.^16^ The few studies that have adopted a longitudinal modelling approach are characterised by several major shortfalls, including failure to enrol an appropriate longitudinal comparison group, selective dropout, very small sample sizes and short periods of follow-up.^15 17 18^

We conducted a longitudinal analysis of the change in cognitive development in EP survivors from infancy to early adulthood in the EPICure study, the largest prospective, population-based cohort of EP births.^2 19^ The main objective of this study was to investigate the cognitive trajectories in EP children compared with those of a term-born comparison group. Our secondary objectives were to examine the impact of sex and maternal education on these trajectories, the two main prognostic factors for cognitive impairment in VP/VLBW children,^20^ and within the EP group the effect of GA and neonatal brain injury, also strongly related to neurodevelopmental outcome.^21–24^

## Methods

### Study population

**Figure 1:**
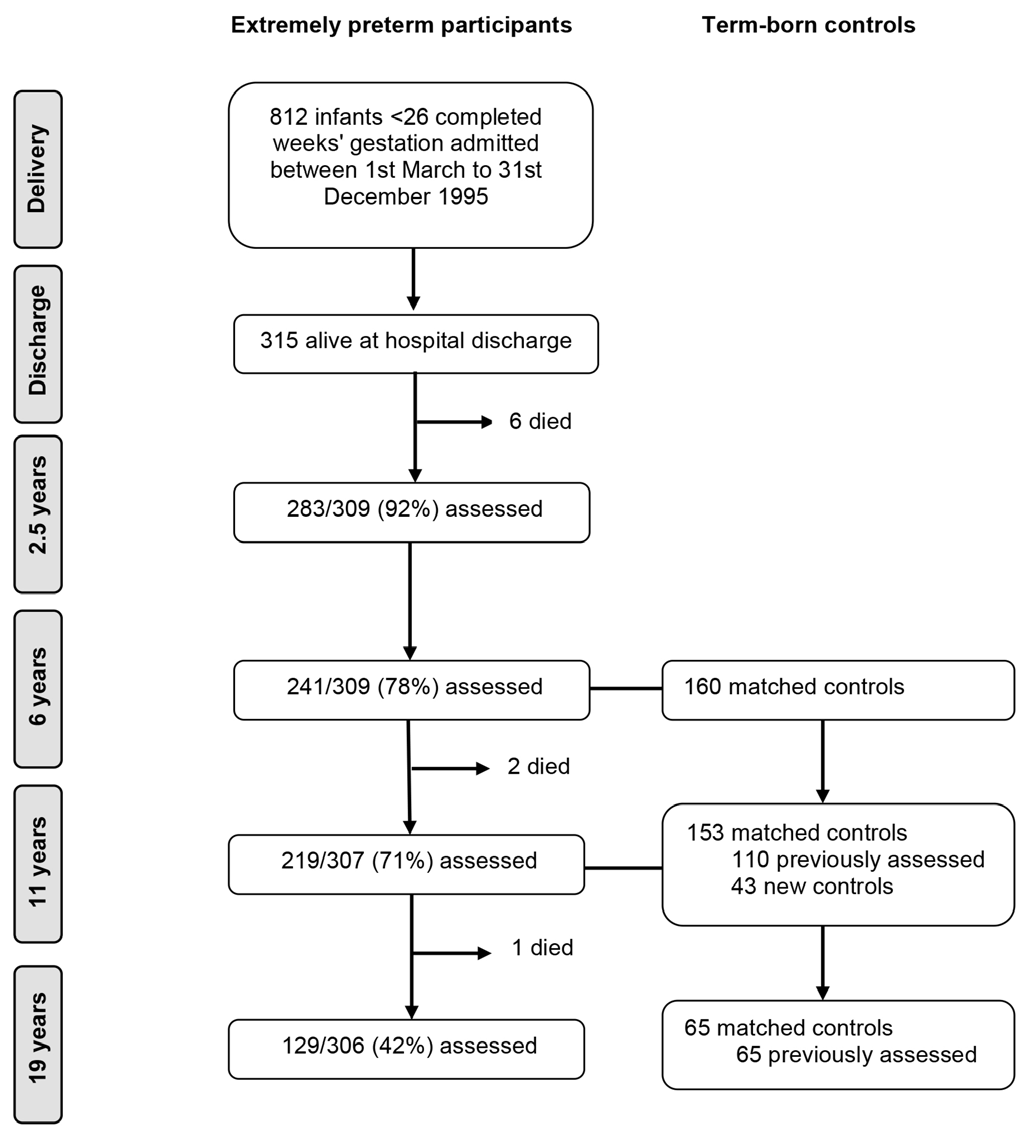
Flow of study participants in the EPICure cohort study.

Recruitment and follow-up to age 11 in the EPICure cohort study have been reported in full previously.^16 25^ All infants born at 25 completed weeks of gestation or less in all 276 maternity units in the UK and Ireland from 1 March through 31 December 1995 were identified. The 315 surviving infants at hospital discharge were invited for assessment at 2.5, 6, 11 and 19 years of age. There were nine deaths between discharge and the 19-year assessment, at which 129/306 (42%) of EP participants were assessed (figure 1). At 6 years, for the 204/241 (85%) children attending mainstream school, a term-born classroom control was identified, matched on age, sex and race. Of the 160 controls assessed at 6 years, 110 (69%) were reassessed at 11 years of age, and 43 replacement controls were identified if the EP child had moved school or the original control declined further participation. At age 19, 65 (42%) of controls evaluated at 11 years participated in assessments.

### Data collection and outcome assessment

Participants were invited for a clinical examination at each time point, including a cognitive assessment. All assessors were blinded to the child’s birth status. Development at age 2.5 years corrected for preterm birth was assessed using the Bayley Scales of Infant Development-Second Edition (BSID-II),^26^ which produces standardised index scores (mean 100; SD 15; lowest score 40) for cognitive development (Mental Development Index; MDI) and motor development (Psychomotor Development Index; PDI). At 6 and 11 years chronological age, the Kaufman Assessment Battery for Children^27^ was used, which yields a mental processing composite (mean 100; SD 15; lowest score 25) score for global cognitive ability. At the 19-year assessment, the Wechsler Abbreviated Scale of Intelligence-Second Edition^28^ was administered, generating a Full Scale IQ score (mean 100; SD 15; lowest score 25).

For some children who attended assessment, it was not possible to administer the planned cognitive test, either due to the child’s behaviour or because of severe neurodevelopmental impairment. However, the category of cognitive ability was estimated according to conventional SD banded classifications, for example 70–84 (−1 to −2SD) or 55–69 (< −3 SD), either based on clinical observation or using an alternative developmental test. Cognitive scores were multiply imputed for 52 individuals at 2.5 years, 42 at 6 years, 18 at 11 years, and 3 at 19 years within the ranges shown in online supplementary table S1. Ten sets of values in the estimated IQ band were imputed and combined to produce overall estimates. No other missing outcome data were imputed for children who were not assessed.

10.1136/archdischild-2017-313414.supp1 Supplementary file 1

The highest maternal educational qualification was recorded over the study period and classified into two groups: O’ level or below, versus A’ level or above (or equivalent). EP children were classified according to the severity of brain injury on their worst scan during the neonatal period: none/mild was defined as no pathology or subependymal/choroidal/intraventricular haemorrhage/ventricular size ≤4 mm over 97th centile with no parenchymal cysts or parenchymal haemorrhage. Moderate/severe was defined as ventricular size >4 mm over 97th centile/haemorrhage/cysts/cystic leucomalacia or any unilateral/bilateral parenchymal problems.

### Statistical analysis

EP participants and term-born controls were classified according to their pattern of missing assessments: completers (no missing assessments) and non-completers (at least one missing assessment). If the participant had attended assessment and a cognitive score was imputed as described above, this was treated as a non-missing value. Maternal and infant characteristics were compared between the completers and non-completers within the EP and control groups. Two-sided P values were calculated using Fisher’s exact test for binary variables and the t-test for continuous variables.

Group mean differences in cognitive scores between EP children and controls were calculated for each time point with 95% CIs. Hierarchical mixed modelling was used to investigate the trajectories of cognitive test scores from infancy to adulthood using Stata/SE V.13.1 for Windows, treating the data as having a hierarchical structure with observations at each time point nested within each individual. This is a sensitive method for assessing change as it can test for different patterns of development (intercept, slope and curvature) and can also incorporate individuals with incomplete data.

In the analysis comparing the EP and control groups, age was fitted as a random effect, which allows both the average level and the change in IQ to vary between individuals. Age was centred at 6 years, when the control group was first assessed, to make the intercept coefficient more meaningful. A group term was added as a fixed covariate to test for a difference in intercept between the EP and control groups. An interaction term between age and group was then added to test whether the EP and control groups varied on slope, and then a quadratic function of age to test for curvature in the trajectories. Next, within-individual variance and between-individual variance were allowed to vary between the EP and control groups by fitting group-specific random intercept and random slope parameters. The effect of participant sex and maternal education was examined by adding them separately to the model as fixed covariates and then as interactions with group. For a parameter to be retained in the model, it was required to have a P value <0.05 in the likelihood ratio test in all 10 multiply imputed data sets. Similar analyses were conducted within the EP group (with the omission of the parameter for group), testing the effect of neonatal brain injury and GA, dichotomised as 25 weeks vs <25 weeks.

Analyses were first conducted in all participants with data available at any time point, and then restricted to completers only.

## Results

### Participants

Baseline characteristics of EP participants and term-born controls by completeness of data are shown in online supplementary table S2 and online supplementary table S3. EP completers were more likely to be from a multiple pregnancy, have mothers who were older, of white ethnicity and better educated, and have fathers with a non-manual occupation. They also had higher BSID-II MDI and PDI scores at 2.5 years and were less likely to have moderate/severe cerebral palsy. There were no statistically significant differences between control participants with complete cognitive assessments and those with at least one missing, except for visual impairment at last assessment (due mainly to prescriptions for glasses at the 19-year assessment, which only 11 non-completers attended).

### Cognitive development in EP individuals and term-born controls

**Figure 2:**
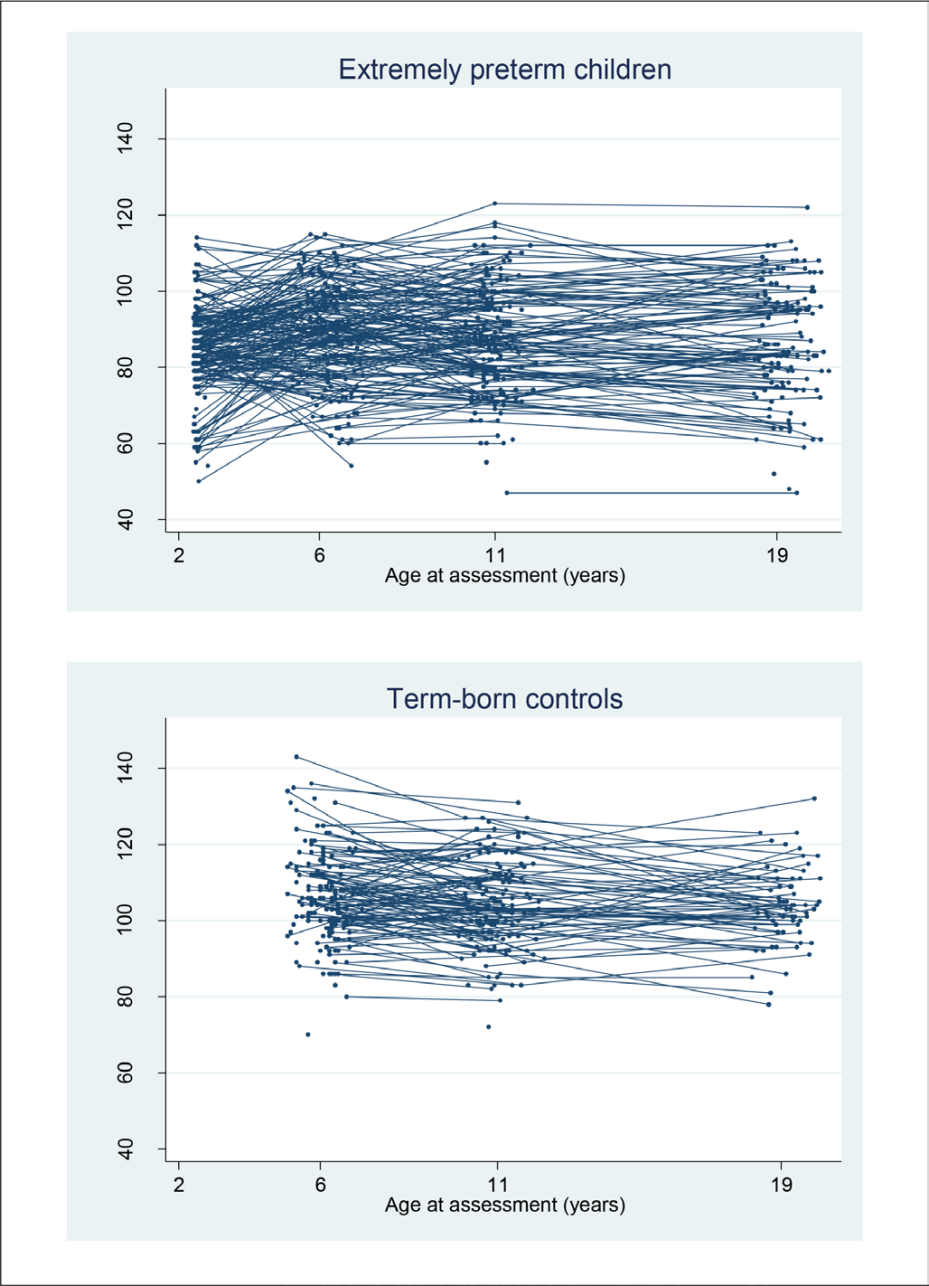
Longitudinal trajectories of observed cognitive test scores in extremely preterm participants and term-born controls from age 2.5 to 19 years.

**Figure 3:**
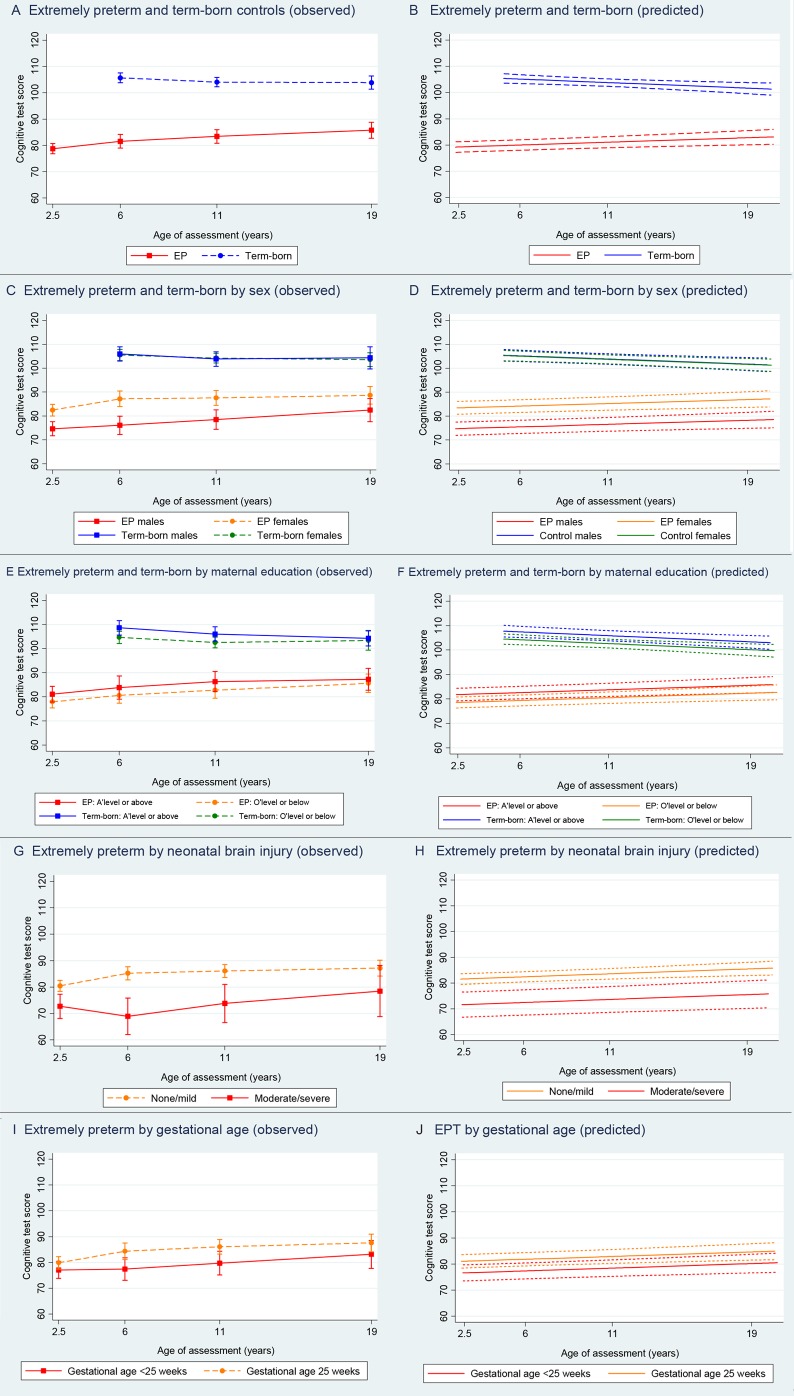
Observed and predicted mean cognitive test scores plus 95% CIs in extremely preterm (EP) participants and term-born controls at ages 2.5, 6, 11 and 19.

Individual trajectories are shown in figure 2, and the mean IQ scores and 95% CIs at each age are presented in table 1. The unadjusted model was based on 495 participants (292 EP and 203 term-born participants) and 1247 IQ scores. The estimated coefficients and 95% CIs are presented in table 2. On average, the predicted IQ scores of EP participants were 25.2 points below their term-born peers (95% CI −27.8 to −22.6, P<0.001). Trajectories were similar between groups, although there was a small but statistically significant increase of 0.5 IQ points per year in the EP group relative to the control group; IQ scores fell by 0.3 points for each year after the age of 6 in the control group and increased by 0.2 points in the EP group. The observed and predicted trajectories of the unadjusted model are displayed in figure 3A,B. The estimated within-individual variation was 5.7 IQ points for control and 8.7 IQ points for EP participants, so the test scores within the EP group at different time points varied more around their average score than in controls. The between-individual variance functions for EP and control participants are displayed in online supplementary figure S1, and indicate that the variance in IQ scores among EP participants is higher than among controls, and increases as they get older.

**Table 1:** Cognitive test scores in extremely preterm (EP) participants and term-born controls by age of assessment, overall and stratified by sex, maternal education, neonatal brain injury and gestational age

	Age 2.5 years	Age 6 years		Age 11 years		Age 19 years	
	EP (n=283)	EP (n=241)	Control (n=160)	EP (n=219)	Control (n=153)	EP (n=127)	Control (n=64)
Cognitive test*	BSID-II MDI	K-ABC MPC		K-ABC MPC		WASI-II FSIQ	
Age at assessment†							
Mean (SD)	2.5 (0.1)	6.3 (0.5)	6.1 (0.5)	11.0 (0.4)	11.0 (0.6)	19.3 (0.6)	19.2 (0.5)
Median (range)	2.5 (2.3–3.3)	6.3 (5.2–7.3)	6.2 (5.1–7.2)	10.9 (10.2–12.2)	10.9 (9.8–12.3)	19.3 (18.4–20.5)	19.2 (18.1–20.1)
**Cognitive test score‡**							
Mean (95% CI)	78.7 (76.7 to 80.7)	81.6 (79.0 to 84.2)	105.7 (103.9 to 107.6)	83.4 (80.8 to 85.9)	104.1 (102.3 to 105.8)	85.7 (82.7 to 88.8)	103.9 (101.4 to 106.4)
Mean difference (95% CI)		−24.2 (−27.7 to −20.6)		−20.7 (−24.1 to −17.3)		−18.1 (−22.8 to −13.5)	
**Infant sex**							
Female							
n (%)	148 (52.3)	119 (49.4)	89 (55.6)	118 (53.9)	89 (58.2)	68 (53.5)	39 (60.9)
Mean (95% CI)	82.4 (80.0 to 84.9)	87.2 (83.9 to 90.4)	105.5 (103.2 to 107.8)	87.6 (84.5 to 90.6)	104.2 (102.2 to 106.3)	88.6 (84.9 to 92.3)	103.6 (100.7 to 106.5)
Male							
n (%)	135 (47.7)	122 (50.6)	71 (44.4)	101 (46.1)	64 (41.8)	59 (46.5)	25 (39.1)
Mean (95% CI)	74.6 (71.7 to 77.6)	76.1 (72.3 to 79.9)	106.0 (103.0 to 109.0)	78.5 (74.4 to 82.6)	103.8 (100.8 to 106.9)	82.4 (77.6 to 87.2)	104.4 (99.7 to 109.0)
Mean difference (95% CI)	−7.8 (−11.6 to −4.0)	−11.1 (−16.2 to −6.1)	0.5 (−3.3 to 4.2)	−9.1 (−14.1 to −4.0)	−0.4 (−4.0 to 3.2)	−6.2 (−12.2 to −0.2)	0.8 (−4.5 to 6.1)
**Maternal education**							
O’ level or below							
n (%)	174 (61.5)	146 (60.6)	82 (51.3)	131 (59.8)	87 (56.9)	64 (50.4)	32 (50.0)
Mean (95% CI)	77.9 (75.4 to 80.4)	80.6 (77.4 to 83.8)	104.7 (102.1 to 107.2)	82.7 (79.4 to 86.1)	102.6 (100.3 to 104.8)	85.6 (81.7 to 89.4)	103.4 (99.4 to 107.3)
A’ level or above							
n (%)	95 (33.6)	83 (34.4)	59 (36.9)	77 (35.2)	58 (37.9)	60 (47.2)	31 (48.4)
Mean (95% CI)	81.1 (77.9 to 84.3)	83.8 (79.0 to 88.7)	108.7 (105.6 to 111.7)	86.3 (82.1 to 90.6)	106.1 (103.0 to 109.1)	87.3 (82.7 to 91.8)	104.3 (101.1 to 107.5)
Mean difference (95% CI)	3.2 (−1.0 to 7.4)	3.2 (−2.4 to 8.9)	4.0 (−0.01 to 8.0)	3.6 (−1.8 to 9.0)	3.5 (−0.2 to 7.2)	1.7 (−4.3 to 7.7)	0.9 (−4.3 to 6.2)
**Neonatal brain injury**							
No/mild brain injury							
n (%)	220 (77.7)	187 (77.6)	160 (100)	169 (77.2)	153 (100)	104 (81.9)	164 (100)
Mean (95% CI)	80.5 (78.3 to 82.6)	85.2 (82.7 to 87.7)	–	86.1 (83.6 to 88.5)	–	87.2 (84.2 to 90.2)	–
Moderate/severe brain injury							
n (%)	63 (22.3)	54 (22.4)	0 (0.0)	49 (22.4)	0 (0.0)	22 (17.3)	0 (0.0)
Mean (95% CI)	72.7 (68.1 to 77.3)	68.9 (62.0 to 75.8)	–	73.8 (66.5 to 81.0)	–	78.4 (68.8 to 88.1)	–
Mean difference (95% CI)	−7.8 (−12.4 to −3.12)	−16.3 (−22.3 to 10.4)	–	−12.3 (−18.3 to −6.3)	–	−8.7 (−16.7 to −0.8)	–
**Gestational age at delivery**							
25 weeks							
n (%)	167 (59.0)	144 (59.8)	0 (0.0)	126 (57.5)	0 (0.0)	75 (59.1)	0 (0.0)
Mean (95% CI)	79.8 (77.5 to 82.2)	84.3 (81.2 to 87.5)	–	86.1 (83.3 to 88.9)	–	87.6 (84.2 to 91.0)	–
<25 weeks							
n (%)	116 (41.0)	97 (40.2)	0 (0.0)	93 (42.5)	0 (0.0)	52 (40.9)	0 (0.0)
Mean (95% CI)	77.1 (73.8 to 80.4)	77.4 (73.1 to 81.8)	–	79.7 (75.1 to 84.4)	–	83.1 (77.7 to 88.6)	–
Mean difference (95% CI)	−2.7 (−6.7 to 1.3)	−6.9 (−12.2 to 1.6)	–	−6.3 (−11.5 to 1.2)	–	−4.5 (−10.6 to 1.7)	–

*BSID-II MDI, Bayley Scales of Infant Development -Second Edition Mental Development Index; K-ABC MPC, Kaufman Assessment Battery for Children  mental processing composite; WASI-II FSIQ, Wechsler Abbreviated Scale of Intelligence-Second Edition – Full Scale IQ.

†Corrected age at year 2.5 and chronological age in years 6, 11 and 19.

‡Values were multiply imputed within a clinically specified range for EP participants seen at assessment but not tested with planned cognitive test: n=52 (year 2.5), n=41 (year 6), n=18 (year 11), n=3 (year 19). Values were multiply imputed for one control in year 6.

**Table 2:** Estimated mean differences in cognitive test scores and 95% CIs from mixed model analyses in extremely preterm participants and term-born controls

Extremely preterm children and term-born classroom controls			
Parameter	Unadjusted model (n=495)	Adjusted for sex (n=495)	Adjusted for maternal education (n=456)
	Estimate (95% CI)	Estimate (95% CI)	Estimate (95% CI)
Fixed			
Constant	105.2 (103.5 to 106.9)	105.1 (103.0 to 107.2)	104.2 (102.2 to 106.3)
EP	−25.2 (−27.8 to −22.6)	−21.0 (−24.4 to −17.6)	−25.0 (−27.7 to −22.2)
Age	−0.3 (−0.5 to −0.1)	−0.3 (−0.5 to −0.1)	−0.3 (−0.5 to −0.1)
EP × age	0.5 (0.2 to 0.7)	0.5 (0.2 to 0.7)	0.5 (0.3 to 0.8)
Male	–	0.1 (−2.8 to 3.0)	–
EP × male	–	−8.8 (−13.6 to −4.0)	–
Higher maternal education	–	–	3.2 (0.8 to 5.7)
Random			
Within-individual			
EP SD	8.7 (8.1 to 9.4)	8.7 (8.1 to 9.4)	8.8 (8.1 to 9.5)
Control SD	5.7 (4.9 to 6.7)	5.7 (4.9 to 6.7)	5.6 (4.8 to 6.6)
Between-individual			
EP intercept SD	16.3 (14.9 to 17.9)	15.8 (14.3 to 17.3)	16.2 (14.7 to 17.9)
EP slope SD	0.5 (0.3 to 0.8)	0.5 (0.3 to 0.8)	0.5 (0.3 to 0.8)
EP corr(intercept, slope)	0.3 (−0.01 to 0.6)	0.4 (0.04 to 0.7)	0.3 (−0.02 to 0.6)
Control intercept SD	10.6 (9.2 to 12.2)	10.6 (9.2 to 12.2)	10.6 (9.1 to 12.2)
Control slope SD	0.7 (0.5 to 1.0)	0.7 (0.5 to 1.0)	0.7 (0.5 to 1.0)
Control corr(intercept, slope)	−0.6 (−0.8 to −0.3)	−0.6 (−0.8 to −0.3)	−0.6 (−0.8 to −0.3)

Extremely preterm children only		
Parameter	Adjusted for neonatal brain injury (n=291)	Adjusted for gestational age (n=292)
	Estimate (95% CI)	Estimate (95% CI)
Fixed		
Constant	82.4 (80.2 to 84.5)	81.8 (79.2 to 84.4)
Age	0.2 (0.06 to 0.3)	0.2 (0.06 to 0.3)
Moderate-severe brain Injury	−10.9 (−15.5 to −6.3)	–
Gestational age <25 weeks	–	−4.4 (−8.4 to −0.4)
Random		
Within-individual SD	8.7 (8.1 to 9.4)	8.7 (8.1 to 9.4)
Between-individual		
Intercept SD	15.6 (14.2 to 17.2)	16.2 (14.7 to 17.7)
Slope SD	0.5 (0.3 to 0.8)	0.5 (0.3 to 0.8)
corr(intercept, slope)	0.3 (−0.03 to 0.6)	0.3 (−0.02 to 0.6)

corr, correlation; EP, extremely preterm.

### Effect of sex and maternal education on cognitive development in EP individuals and term-born controls

Adding sex as a main effect to the unadjusted model did not have significant effect, but there was evidence of an interaction between sex and group (table 2). The estimated IQ scores of EP boys were on average 8.8 points below EP girls (95% CI −13.6 to −4.0, P<0.001), but there was no significant difference between boys and girls in the control group. The estimated IQ scores of participants with more highly educated mothers were 3.2 points higher in both the EP and control groups (95% CI 0.8 to 5.7, P=0.01). The interaction between education and group was not significant, implying that the impact of maternal education on the rate of cognitive development was similar in both groups (table 2). Observed and predicted mean trajectories adjusted for sex and maternal education are displayed in figure 3C–F.

### Effect of neonatal brain injury and GA on cognitive development within EP individuals

The mean cognitive test scores and 95% CIs at each age for EP participants, stratified by severity of neonatal brain injury and GA, are presented in table 1. In the predicted model (table 2), scores of EP participants with moderate/severe neonatal brain injury were on average 10.9 points below participants with no/mild brain injury (95% CI −15.5 to −6.3, P<0.001). The interaction term between brain injury and age was not significant, indicating that the rate of cognitive development between those with none/mild and moderate/severe brain injury was similar over time. On average, the IQ scores of participants born less than 25 weeks’ gestation were 4.4 points lower than participants born at 25 weeks (95% CI −8.4 to −0.4, P=0.03), and the interaction between GA and age was also not significant. The observed and predicted mean trajectories adjusted for neonatal brain injury and GA are displayed in figure 3G–J.

### Complete-case analysis

All analyses were repeated for the 167 completers (114 EP and 63 controls) and the results are shown in online supplementary table S4 and figure S2. IQ scores were around 3 points higher on average in the complete-case analysis compared with the analysis including all participants. All of the differences reported between groups were of similar magnitude in the complete-case analysis, and the same results were statistically significant, except for a reduced effect of maternal education: difference in means for completers: 2.1 points (95% CI −1.6 to 5.9, P=0.26).

## Discussion

In this large dual-nation, population-based study, we found that cognitive trajectories were similar between groups, both stable over time, with persistent deficit in the EP group into early adulthood. IQ scores were on average 25 points lower in EP individuals, with no evidence of substantial ‘catch-up’ with term-born peers, although the deficit closed slightly by 0.5 IQ points each year, amounting to 6.5 points over the study period. Scores were also more variable both within individuals and between individuals in the EP group. While there were no differences between term-born boys and girls, being an EP boy had a detrimental effect on cognitive function, with scores over 8 points lower on average than EP girls, persisting across childhood and adolescence. There was some evidence that higher maternal education was marginally beneficial in both the EP and control groups. Having moderate to severe neonatal brain injury also had an adverse effect on outcome for EP individuals, who had IQ scores 10 points lower on average compared with EP individuals with no/mild brain injury. Survivors born at 25 weeks of gestation had significantly better cognitive function than those born less than 25 weeks, by a magnitude of about 5 points.

Our findings in this contemporary EP cohort followed prospectively from birth were consistent with those from older VP/VLBW cohorts in which cognitive scores were persistently lower by around 10 points compared with term-born controls into early adulthood. There is thus a significant decline in cognitive function with lower gestation as has been shown in childhood.^29^ The association between the level of maternal education and socioeconomic status and cognitive outcome in both term and preterm populations has been frequently reported,^8 30^ yet there was only weak evidence of this association in the present study. This may be due to the higher dropout rate in younger, less educated mothers, which restricted the educational range included in the analysis, making it less sensitive to detect a difference. It is also possible that maternal education alone is not a sufficient marker of social disadvantage. The impact of sex on the cognitive trajectory of EP participants supports the findings from cross-sectional studies that have shown that EP boys are inherently disadvantaged and at greater risk of poorer neurodevelopmental outcome than their female counterparts.^31 32^ We have shown that this effect persists into adulthood and that EP boys do not catch up with EP girls. The adverse effect of neonatal brain injury on subsequent cognitive function is well documented by studies that report strong linear trends with severity of brain injury.^21 23^ Widespread structural changes are still seen in adulthood and have been shown to be associated with IQ.^33^ The larger between-individual differences among EP participants may be partly explained by factors such as sex and brain insult. The larger within-individual variance was mainly driven by the lack of control data at 2.5 years; participants with severe cognitive impairment were more likely to have greater variation in scores over time due to the difference in the lower limits of the BSID-II and the later IQ tests.

In common with other longitudinal studies, the numbers of participants lost to follow-up increased over time and were related to markers of social disadvantage and disability. It is therefore likely that we have underestimated the true extent of cognitive impairment in the EP group, particularly at older ages. It is not certain whether selective dropout explains the small but significant increase in IQ scores over time in the EP group because a similar increase was observed in the complete-case analysis. The classroom controls recruited from mainstream schools are likely to represent a relatively healthy group, and therefore any differences found may have been exaggerated. However, we believe this to be minimal, as most children with special educational needs in the UK are integrated into mainstream schools and only 1.1% of children have special-school placements.^34^ Hence the inclusion of classmates for EP participants attending special schools (15% in this study) would have inappropriately biased the comparison group. Importantly, our findings were strengthened by the analysis of individuals with complete follow-up, which corroborated the main results. Although this subset of participants had slightly higher cognitive scores in general due to the higher rate of dropout in those with lower developmental scores in infancy, the same differences between groups were detected and were of similar magnitude.

In conclusion, it appears that being born EP places limits on brain plasticity and function with very little recovery over time, with the most vulnerable being male and those who experienced brain injury early in life. Acquired brain lesions during the neonatal period have been shown to cause focal and diffuse structural abnormalities, which may disturb neurodevelopmental processes and impede the brain from maintaining a normal developmental trajectory.^35 36^ If EP participants fail to achieve optimum levels of cognitive function once they have reached maturity, then this has implications for health and well-being in later adulthood and old age.^37^
