# Supplementary material for: Cognitive trajectories from infancy to early adulthood following birth before 26 weeks of gestation: a prospective, population-based cohort study
Source: Arch Dis Child. 2017 Nov 16;103(4):363–70. doi: 10.1136/archdischild-2017-313414 (PMC5890637; doi:10.1136/archdischild-2017-313414)
Supplement: Supplementary file 1 [file archdischild-2017-313414supp001.docx]

**Supplementary Material**

**Contents**

[Table S1: Summary of imputed cognitive test scores by age of assessment 1](#_Toc482717587)

[Table S2: Perinatal and neurodevelopmental characteristics of extremely preterm participants according to completeness of follow-up assessment 2](#_Toc482717588)

[Table S3: Characteristics of term-born controls according to completeness of follow-up assessment 4](#_Toc482717589)

[Figure S1: Predicted between-individual variance function for extremely preterm participants and term-born controls 5](#_Toc482717590)

[Table S4: Complete case analysis: estimated mean differences in cognitive test scores and 95% confidence intervals from mixed model analyses in extremely preterm participants and term-born controls 6](#_Toc482717591)

[Figure S2: Complete case analysis: observed and predicted mean cognitive test scores plus 95% confidence intervals in extremely preterm participants and term-born controls at age 2.5, 6, 11 and 19 7](#_Toc482717592)

# Table S1: Summary of imputed cognitive test scores by age of assessment

| **Imputed range for cognitive test score** | **No. (%) of extremely preterm participants with imputed values by assessment year** | | | | | | | | |
| --- | --- | --- | --- | --- | --- | --- | --- | --- | --- |
|  | **2.5 years**  (n=283) | | | **6 years**  (n=241) | | **11 years**  (n=217) | | **19 years**  (n=127) | |
| 85-114 | 2 |  | 2^a^ | |  | 2 |  | - |  |
| 70-84 | 5 |  | 5 | |  | - |  | - |  |
| 55-69 | 10 |  | 9 | |  | - |  | - |  |
| 40-54 | 35 |  | 7 | |  | - |  | - |  |
| 25-39 | - |  | 19 | |  | 16 |  | 3 |  |
| Total | 52 | (18.4) | 42 | | (17.4) | 18 | (8.3) | 3 | (2.4) |
| ^a^ One control also had imputed values in the range 85-100 at age 6 years. | | | | | | | | | |

# Table S2: Perinatal and neurodevelopmental characteristics of extremely preterm participants according to completeness of follow-up assessment

|  | **Completers:**  **4 assessments**  **(n=114)** | | **Non-completers**  **<4 assessments**  **(n=201)** | ***P* value^a^** |
| --- | --- | --- | --- | --- |
| **Maternal/paternal factors**  Maternal age (years), mean [SD]: | 29.4 [5.4] (n=113) | | 27.9 [6.1] (n=200) | 0.03 |
| Mother of non-white ethnicity, % (n): | 15.9% (18/113) | | 27.4% (55/201) | 0.03 |
| Primigravida, % (n): | 35.1% (40/114) | | 27.5% (55/200) | 0.16 |
| Mother smoked during pregnancy, % (n): | 27.4% (31/113) | | 36.9% (62/168) | 0.12 |
| Antenatal steroids given, % (n): | 81.6% (93/114) | | 77.4% (154/199) | 0.47 |
| Mother has A’ level or above (or equivalent), % (n):^b^ | 49.6% (55/111) | 27.0% (44/163) | | <0.001 |
| Father's occupation non-manual, % (n):^b^ | 51.0% (50/98) | 26.5% (35/132) | | <0.001 |
| **Infant perinatal factors**  Multiple birth, % (n): | 32.5% (37/114) | 21.0% (42/200) | | 0.03 |
| Congenital anomaly present, % (n): | 1.8% (2/114) | 3.0% (6/200) | | 0.72 |
| Gestational age 24 weeks or less, % (n): | 43.0% (49/114) | 39.8% (80/201) | | 0.63 |
| Birthweight (grams), mean [SD]: | 745 [127] (n=114) | 749 [107] (n=201) | | 0.76 |
| Male sex, % (n): | 45.6% (52/114) | 51.2% (103/201) | | 0.35 |
| Moderate/Severe brain injury during neonatal period, % (n):^c^ | 18.4% (21/114) | 25.0% (50/200) | | 0.21 |
| Laser or cryotherapy for retinopathy of prematurity, % (n): | 14.3% (16/112) | 14.7% (29/198) | | 1.00 |
| Laparotomy for necrotising enterocolitis, % (n): | 1.8% (2/111) | 3.6% (7/197) | | 0.50 |
| Supplemental oxygen at 36 weeks, % (n): | 71.1% (81/114) | 75.6% (152/201) | | 0.42 |
| **Infant developmental outcomes at 2.5 years** |  |  | |  |
| BSID-II Mental Developmental Index, mean [SD]: | 82.7 [14.4] (n=114) | 76.1 [17.2] (n=169) | | 0.001 |
| BSID-II Psychomotor Developmental Index, , mean [SD]: | 83.9 [17.4] (n=108) | 79.0 [18.6] (n=154) | | 0.03 |
| BSID-II Behaviour Rating Percentile, , mean [SD]: | 36.4 [26.6] (n=108) | 35.7 [24.5] (n=151) | | 0.82 |
| CBCL Total Problem T-score, mean [SD]: | 55.6 [9.4] (n=112) | 55.8 [9.9] (n=158) | | 0.89 |
| **Neurodevelopmental impairment at last assessment^b^**  Cerebral palsy, % (n): | 8.8% (10/114) | 23.8% (46/193) | | 0.001 |
| Moderate/severe cerebral palsy, % (n): | 6.1% (7/114) | 14.7% (28/190) | | 0.03 |
| Moderate/severe cognitive impairment, % (n): | 45.6% (52/114) | 49.4% (88/178) | | 0.55 |
| Moderate/severe visual impairment, % (n): | 7.9% (9/114) | 12.5% (24/192) | | 0.26 |
| Moderate/severe hearing impairment, % (n): | 0.9% (1/114) | 2.6% (5/193) | | 0.42 |
| Moderate/severe functional impairment, % (n):^d^ | 47.4% (54/114) | 53.9% (96/178) | | 0.28 |
| Severe functional impairment, % (n):^d^ | 20.2% (23/114) | 27.5% (49/178) | | 0.17 |
| ^a^ Two-sided p-values were calculated using Fisher's Exact Test for binomial variables and the t-test for continuous variables.  ^b^ Not collected at discharge; first collected at one or two year assessment.  ^c^ Parenchymal pathology and/or ventriculomegaly on worst cranial ultrasound scan before discharge home.  ^d^ Functional impairment includes cerebral palsy, cognitive, visual or hearing impairment. | | | | |

# Table S3: Characteristics of term-born controls according to completeness of follow-up assessment

|  | **Completers:**  **3 assessments**  **(n=53)** | **Non-completers**  **<3 assessments**  **(n=153)** | ***P* value^a^** |
| --- | --- | --- | --- |
| Mother has A ’level or above (or equivalent), % (n): | 48.1% (25/52) | 37.9% (50/132) | 0.24 |
| Male sex, % (n): | 39.6% (21/53) | 43.8% (67/153) | 0.63 |
| Any cognitive impairment at last assessment, % (n) | 17.0% (9/53) | 18.7% (28/150) | 0.84 |
| Moderate/severe cognitive impairment at last assessment, % (n): | 3.8% (2/53) | 2.0% (3/150) | 0.61 |
| Any visual impairment at last assessment, % (n): | 39.6% (21/53) | 14.1% (21/149) | <0.001 |
| Any hearing impairment at last assessment, % (n): | 1.9% (1/53) | 0.0% (0/149) | 0.26 |
| Any functional impairment at last assessment, % (n):^b^ | 49.1% (26/53) | 30.9% (46/149) | 0.02 |
| ^a^ Two-sided p-values were calculated using Fisher's Exact Test.  ^b^ Functional impairment includes cerebral palsy, cognitive, visual or hearing impairment. | | | |

# Figure S1: Predicted between-individual variance function for extremely preterm participants and term-born controls

| **** |
| --- |
| Variance function for EP group = 16.3^2^ + 2 x 2.7(Age-6) + 0.5^2(^Age-6)^2^  Variance function for control group = 10.6^2^ - 2 x 4.2(Age-6) + 0.7^2^(Age-6)^2^  The variance in cognitive test scores between EP participants is higher than among controls and increases as they get older, whereas the between-individual variance dips slightly in the control group. The mean difference in intercepts is lower in control participants (10.6 versus 16.3 points), but the mean difference in slopes is higher (0.7 versus 0.5 points). |

# Table S4: Complete case analysis: estimated mean differences in cognitive test scores and 95% confidence intervals from mixed model analyses in extremely preterm participants and term-born controls

| **Extremely preterm participants and term-born classroom controls** | | | | | | | |
| --- | --- | --- | --- | --- | --- | --- | --- |
| **Parameter** | | **Unadjusted model**  **(n=167)** | | **Adjusted for sex**  **(n=167)** | | **Adjusted for maternal education (n=163)** | |
|  |  | **Estimate** | **95% CI** | **Estimate** | **95% CI** | **Estimate** | **95% CI** |
| **Fixed** | |  |  |  |  |  |  |
| Constant | | 108.2 | (105.1 to 111.3) | 108.5 | (104.8 to 112.2) | 107.4 | (103.9 to 111.0) |
| EP | | -23.9 | (-28.1 to -19.8) | -19.5 | (-24.5 to -14.4) | -23.7 | (-27.8 to -19.6) |
| Age | | -0.4 | (-0.6 to -0.2) | -0.4 | (-0.6 to -0.2) | -0.4 | (-0.6 to -0.2) |
| EP*Age | | 0.5 | (0.2 to 0.8) | 0.5 | (0.2 to 0.8) | 0.6 | (0.3 to 0.8) |
| Male | | - | - | -0.8 | (-5.9 to 4.4) | - | - |
| EP*Male | | - | - | -9.7 | (-17.1 to -2.4) | - | - |
| Higher maternal education | | - | - | - | - | 2.1 | (-1.6 to 5.9) |
|  | |  |  |  |  |  |  |
| **Random** | |  |  |  |  |  |  |
| Within-individual | |  |  |  |  |  |  |
| EP sd | | 7.5 | (6.8 to 8.3) | 7.5 | (6.9 to 8.4) | 7.5 | (6.8 to 8.3) |
| Control sd | | 4.8 | (4.0 to 5.8) | 4.8 | (4.0 to 5.8) | 4.8 | (4.0 to 5.8) |
| Between-individual | |  |  |  |  |  |  |
| EP Intercept sd | | 14.3 | (12.4 to 16.6) | 13.3 | (11.5 to 15.4) | 13.7 | (11.8 to 15.9) |
| EP Slope sd | | 0.6 | (0.5 to 0.8) | 0.6 | (0.5 to 0.8) | 0.6 | (0.5 to 0.8) |
| EP corr(intercept, slope) | | 0.1 | (-0.2 to 0.4) | 0.1 | (-0.2 to 0.4) | 0.1 | (-0.2 to 0.3) |
| Control Intercept sd | | 10.7 | (8.5 to 13.3) | 10.6 | (8.5 to 13.3) | 10.5 | (8.4 to 13.2) |
| Control Slope sd | | 0.7 | (0.5 to 0.9) | 0.7 | (0.5 to 0.9) | 0.7 | (0.5 to 0.9) |
| Control corr(intercept, slope) | | -0.5 | (-0.7 to -0.2) | -0.5 | (-0.7 to -0.2) | -0.5 | (-0.7 to -0.2) |
| **Extremely preterm participants only** | | | | | | | |
| **Parameter** | **Adjusted for neonatal**  **brain injury (n=114)** | | | **Adjusted for gestational age (n=114)** | |  | |
|  | **Estimate** | | **95% CI** | **Estimate** | **95% CI** |  | |
| **Fixed** |  | |  |  |  |  | |
| Constant | 86.1 | | (83.2 to 89.1) | 87.1 | (83.5 to 90.7) |  | |
| Age | 0.1 | | (-0.02 to 0.3) | 0.1 | (-0.02 to 0.3) |  | |
| Moderate-severe brain Injury | -10.1 | | (-16.9 to -3.2) | - | - |  | |
| Gestational age <25 weeks | - | | - | -6.6 | (-12.0 to -1.2) |  | |
|  |  | |  |  |  |  | |
| **Random** |  | |  |  |  |  | |
| Within-individual sd | 7.5 | | (6.9 to 8.3) | 7.5 | (6.9 to 8.3) |  | |
| Between-individual |  | |  |  |  |  | |
| Intercept sd | 13.8 | | (11.9 to 15.9) | 14.0 | (12.1 to 16.1) |  | |
| Slope sd | 0.6 | | (0.5 to 0.8) | 0.6 | (0.5 to 0.8) |  | |
| corr(intercept, slope) | 0.1 | | (-0.2 to 0.4) | 0.1 | (-0.2 to 0.4) |  | |
| Abbreviations: EP extremely preterm; sd standard deviation; corr correlation; CI confidence interval. | | | | | | | |

# Figure S2: Complete case analysis: observed and predicted mean cognitive test scores plus 95% confidence intervals in extremely preterm participants and term-born controls at age 2.5, 6, 11 and 19

| **** | **** |
| --- | --- |
| **** | **** |
| **** | **** |
| **** | **** |
| **** | **** |
